# 3D monitoring of the surface slippage effect on micro-particle sedimentation by digital holographic microscopy

**DOI:** 10.1038/s41598-021-92498-0

**Published:** 2021-06-21

**Authors:** Majid Panahi, Ramin Jamali, Vahideh Farzam Rad, Mojtaba Khorasani, Ahamd Darudi, Ali-Reza Moradi

**Affiliations:** 1 Department of Physics, Faculty of Science, University of Zanjan, Zanjan, 45371-38791 Iran; 2 Department of Physics, Institute for Advanced Studies in Basic Sciences (IASBS), Zanjan, 45137-66731 Iran; 3 Department of Chemistry, Institute for Advanced Studies in Basic Sciences (IASBS), Zanjan, 45137-66731 Iran; 4 Research Center for Basic Sciences and Modern Technologies (RBST), Institute for Advanced Studies in Basic Sciences (IASBS), Zanjan, 45137-66731 Iran; 5 School of Nano Science, Institute for Research in Fundamental Sciences (IPM), Tehran, 19395-5531 Iran

**Keywords:** Imaging and sensing, Optofluidics

## Abstract

In several phenomena in biology and industry, it is required to understand the comprehensive behavior of sedimenting micro-particles in fluids. Here, we use the numerical refocusing feature of digital holographic microscopy (DHM) to investigate the slippage effect on micro-particle sedimentation near a flat wall. DHM provides quantitative phase contrast and three-dimensional (3D) imaging in arbitrary time scales, which suggests it as an elegant approach to investigate various phenomena, including dynamic behavior of colloids. 3D information is obtained by post-processing of the recorded digital holograms. Through analysis of 3D trajectories and velocities of multiple sedimenting micro-particles, we show that proximity to flat walls of higher slip lengths causes faster sedimentation. The effect depends on the ratio of the particle size to (1) the slip length and (2) its distance to the wall. We corroborate our experimental findings by a theoretical model which considers both the proximity and the particle interaction to a wall of different hydrophobicity in the hydrodynamic forces.

## Introduction

Investigating the behavior of small particles’ motion in fluids involves a wide range of phenomena. These phenomena are intensively interested in scientists and engineers in various fields such as physics, biology, geology, and chemical engineering. Several processes for the separation of particles and, accordingly, designing of the particle separation equipment are based on the motion of particles and their control mechanisms in fluids. Sedimentation of particles in fluids, i.e., the motion of particles under the action of gravity field is an effective process in this regard, in which particles may move together. Understanding the behavior of sedimenting particles is important for fundamental studies and various applications such as blood flow, microfluidics, membrane transport, fluidized beds, separation in multiphase systems, oil-mineral aggregate formation, determining micro-particle porosity, color, gravity-driven drainage of paints, coating, cosmetic and ceramic processing^1–5^.

Apart from the gravitational force, the hydrodynamic interactions, the Brownian motion and the inherent motility of particles, and the effect of boundaries are some of the processes and parameters that must be considered in the investigation of a particle’s sedimentation behavior, especially in microscopic scales. Among these processes and parameters, the boundaries’ proximity due to its effect on the overall hydrodynamic interactions and the resulting motion of the sedimenting particles in a quiescent fluid has attracted high attention^6,7^. Studies show that the level of proximity influence depends on factors such as the geometry of the wall, particle size and shape, the particle-wall distance, and the Newtonian/nonNewtonian characteristics of the fluid^8,9^.

The earliest relevant work in understanding and modeling hydrodynamic forces on a particle in motion near a flat wall was done by Goldman et al. in 1967 in two seriate papers, where the interaction between a sphere and a wall in a linear shear flow studied theoretically^10,11^. Other related works include the study of the axisymmetric sedimentation of spherical particles in a viscoelastic fluid^12^, the study of the motion of a spherical particle along a rough wall^13^, the study of sedimentation of a sphere near a vertical wall in an Oldroyd-B fluid^14^, and numerical simulations of a shear flow past a spherical particle sitting over a rough wall^15^.

Although most studies of particle-wall hydrodynamic behavior have been carried out on the macro-scale and in the field of geology^4^, the micro-scale understanding of its interactions in complex fluids and advanced materials technologies is important and includes unanswered questions^16^. Recently, using digital holographic microscopy (DHM), we studied the proximity effect of a flat wall on the sedimentation of spherical colloids in a Newtonian fluid^17^ and the sedimentation of colloidal micro-particles and red blood cells in non-Newtonian fluids^18^. Here, by extracting the 3D trajectories of multiple sedimenting micro-particles from the DHM results, we investigate another important issue on the behavior of sedimentation of micro-particles close to a flat wall: the effect of slippage.

Throughout the study, we consider the micro-particles to sediment near distances to the wall so that they feel the boundary presence. The diffusion coefficient of a particle close to a flat wall is smaller than a particle far from the wall. The reason is the increase in the Stokes drag force^19^. The standard boundary condition for fluid flow along a wall is a no-slip condition. If the wall is at rest, the tangential fluid velocity at the wall vanishes. However, for most practical situations, such as water flowing in thin capillaries, small evidence for slippage is expected^20^. Apart from the dominating size-effect, a great number of interfacial properties have found to affect slip behavior, such as wall wettability and roughness, stiffness, and lattice orientation, as well as other flow properties, such as fluid density, forces that drive the flow, shear rate, and temperature^21^.

In^15^, it is shown by numerical simulations, that hydrodynamic forces acting on a particle falling in the boundary layer of a wall and the resulting motion of the particle are greatly influenced by the wall morphology. Here, we experimentally quantify the effect of wall morphology on the sedimenting micro-particles. DHM is an elegant approach for quantitative visualizing the distributed micro-particles in a volume of a fluid. DHM in transmission mode, in general, is suited well for non-destructive and quantitative visualization of “phase” objects. Several important specimens, including biological samples, are in this category, and since its development, upon advancing the digital recording devices and computers, it has been used extensively for various researches in physics, biology, chemistry, and material sciences, along with several medical and industrial applications^22–24^. DHM is the combination of conventional microscopy and laser interferometry. The recorded interference pattern between the scattered light from the specimen and a reference beam is recorded on a digital sensor, which is called a digital hologram. The recorded digital holograms are subjected to numerical reconstruction, which resembles numerically the diffraction and propagation procedures of conventional holography, and provides whole-field information about the object^22–25^. Therefore, according to the digital nature of the method, it may provide real-time 3D imaging data at time scales from milliseconds to hours, justifying it as a suitable approach to study dynamic phenomena. Moreover, due to its optics-basis and single-exposure nature, DHM is capable to be integrated with other optical methodologies such as other imaging and manipulation techniques^25–27^.

The distribution of identical micro-particles inside a bulk of a transparent fluid can also be considered as a phase object. However, it can be better understood to consider the case as several objects in various depths. Then, by using the numerical refocusing feature of DHM, in the post-processing of the digital holograms, it is possible to find the exact axial position of several objects from a single digital hologram. The lateral position can be simply derived from the intensity image. Therefore, complete information of the position of multiple objects in the field of view will be obtained from a single hologram, and if the procedure is applied on successively recorded digital holograms, the system will be highly suited for the investigation of a broad class of phenomena that involve dynamics of a collection of micro-objects in bulk of the fluid.

To acquire 3D images of microscopic objects, several methodologies have been developed and used. Some of them are based on electrical, acoustical, or mechanical scanning, such as confocal microscopy, scanning electron microscopy, atomic force microscopy, etc.^28–30^. While such methods are beneficial for a wide class of important samples, however, their scanning nature avoids use for dynamic samples. In recent years, instead, another class of methods have been considered, which points, not fully imaging of the sample, but to the tracking of several micro-objects of preferably identical shapes. Some of these methods are designed according to the requirement and limitations of the specific applications, for example, in the case of monitoring particles in non-transparent fluids^31^ or when the particles have substantial absorption at special wavelengths^5,12^. One possibility to this end was stereoscopic approaches, which come along with severe imaging constraints, e.g., small viewing angle, and adjustment problem^32^. Astigmatic particle tracking velocimetry is a particle image-based and single-camera method to provide 3D trajectories of suspended in a fluid and has been used in acoustofluidics, electroosmotic flows, electrochemistry, and electrothermal flows^33–35^. This approach uses cylindrical lenses and, by observing the non-circular shape of the out-of-focus objects, estimates the depth information of the object. However, this technique requires a robust and precise calibration and maintenance of the experimental conditions. In-line holography is another alternative; however, it has insufficient accuracy for the out-of-plane displacement measurements^36,37^. For particle tracking and velocimetry, further approaches can be named, such as deconvolution microscopy, depth coding via three pinholes, correlation evaluation algorithms, etc., and all have their own benefits and drawbacks^38,39^. It is also remarkable that an important step ahead for most of the aforementioned methods has been enhancing their accuracy through the incorporation of deep neural networks, particularly for heterogeneous and crowded samples^40^.

DHM in off-axis mode, on the one hand, is not a scanning-based method; therefore, it is a suitable approach for dynamic phenomena, including the ones in microfluidics. On the other hand, not only its numerical refocusing feature can be used for tracking multiple particles at once, but also, at the same time, it is able to acquire quantitative images of the samples, which is an important advantage over tracking approaches. Therefore, for example, by analysis of successive holograms, the reshaping of moving non-rigid particles in a fluid can be 3D imaged, and their movement can be tracked at the same time^18^. Taking into account these features, DHM is the chosen approach in this research. In^41^, the various features of DHM capabilities for 3D particle tracking is reviewed.

## Theoretical model

Stokes’ law describes the hydrodynamic drag force on a sphere moving in an unbounded quiescent low Reynolds number fluid as:

1 $$\begin{aligned} F_{0}=-6 \pi \eta a v_{0}, \end{aligned}$$

where $\eta$ is the fluid viscosity, and *a* and $v_{0}$ are the radius and the velocity of the moving sphere, respectively. The Reynolds number of the fluid used in this research is approximately 10 $\upmu$s), for which the Eq. (1) is valid. In the sedimentation process, the gravitational force causes the particles to fall, which leads to a terminal velocity when the gravitational force balances the drag force. The terminal velocity can be obtained by Eq. (1). In the presence of a boundary, such as a flat wall, the hydrodynamic forces can also apply on a moving particle. However, for particles larger than a few micrometers, these forces can be neglected with respect to the gravitational force, and therefore, the motion can only be considered in the gravitation direction that is parallel to the flat wall^42^.

It is shown that when a particle sediments in proximity to a flat wall, its sedimenting velocity is hindered due to the increase in the drag force^10,43^. The proximity effect on the motion of micro-particles has been experimentally validated for both Newtonian and non-Newtonian cases^17,18^. Indeed, the proximity effect multiplies a correction factor ($\digamma _{h}^{-1}$) to the drag force. $\digamma _{h}^{-1}$ using “the method of reflection” for a particle with radius *a* which falls at a distance *h* from the no-slip flat wall is represented as a power series of $\frac{a}{h}$^8^:

2 $$\begin{aligned} \digamma _{h}^{-1} \cong 1-\frac{9}{16} \Big (\frac{a}{h}\Big )+\frac{1}{8} \Big (\frac{a}{h}\Big )^{3}-\frac{45}{256} \Big (\frac{a}{h}\Big )^{4}-\frac{1}{16} \Big (\frac{a}{h}\Big )^{5}+O \Big (\frac{a}{h}\Big )^{6}. \end{aligned}$$

In larger $\frac{h}{a}$, $\digamma _{h}^{-1}$ approaches 1, and the conventional Stokes’ law is valid. On the other hand, in very small $\frac{h}{a}$, the drag force increases dramatically. Previous researches on sphere sedimentation have been performed mostly in near a no-slip flat wall. Here, we address the particle sedimentation near a slip flat wall. We investigate this phenomenon in different hydrophobicities. It is the combination of a very large contact angle and a low hysteresis that defines a surface with slippage, and this is because such surfaces are able to trap air at the liquid-solid interface^44^.

The hydrodynamic interaction of a particle with a slip surface is relevant to several applications such as atomic force microscope, dynamic force experiments, and coagulation phenomenon. The slip surfaces reduce drag, and hydrodynamic interaction with a slip flat wall is weaker than with a no-slip flat wall^45,46^. This reduction may be expressed by multiplying a correction factor, $\digamma _{t}$, to drag force for a micro-particle falling close to a slip flat wall^47^:

3 $$\begin{aligned} F_{t}=-6 \pi \mu a v_{t} \digamma _{t}, \end{aligned}$$

where $v_{t}$ is the velocity of the sedimenting particle. The drag force is replaced by $\Delta \rho Vg$, where $\Delta \rho$ is the difference between the densities of the particle and the surrounding medium, *V* is the particle volume, and *g* is the gravitational acceleration constant^17^. The hindered velocity of the sedimenting particle can be, therefore, obtained as:

4 $$\begin{aligned} v_{t}=\frac{\Delta \rho Vg}{6 \pi \mu a v_{t} \digamma _{t}}=\digamma _{t}^{-1} v_{0}, \end{aligned}$$

where the correction factor for this case is^47^:

5 $$\begin{aligned} \digamma _{t}^{-1}=1+\frac{3}{8}\frac{a}{h}\bigg [1-2{\mathcal {J}}(\frac{h}{b})+O\Big (\frac{a}{h}\Big )^{3}\bigg ], \end{aligned}$$

in which *b* is the slip length, and ${\mathcal {J}}$ is a function given by:

6 $$\begin{aligned} {\mathcal {J}}(X)=-\frac{X}{8}(X+3)+\frac{X}{8}(X+2)^{2}\exp (X)Ei(X)+2X\exp (2X)Ei(2X). \end{aligned}$$

In Eq. (6), *Ei* denotes the exponential integral function. This function cannot be calculated expressively. However, by numerical calculation, an approximate can be obtained, from which the correction factor can be written as^46^:

7 $$\begin{aligned} \digamma _{t}^{-1}= & {} 1+\frac{9}{16} \Big (\frac{a}{h}\Big )+[\frac{81}{256}-\frac{9{\bar{b}}}{16}]\Big (\frac{a}{h}\Big )^{2}+[\frac{217}{4096}-\frac{81{\bar{b}}}{128}+\frac{9{\bar{b}}^{2}}{8}]\Big (\frac{a}{h}\Big )^{3}+{} \nonumber \\&{} +[\frac{8865}{65536}-\frac{651{\bar{b}}}{4096}+\frac{405{\bar{b}}^{2}}{256}-\frac{81{\bar{b}}^{3}}{16}]\Big (\frac{a}{h}\Big )^{4}+{} \nonumber \\&{}+[\frac{207529}{1048576}-\frac{8865{\bar{b}}}{16384}-\frac{2655{\bar{b}}^{2}}{4096}-\frac{891{\bar{b}}^{3}}{128}+\frac{261{\bar{b}}^{4}}{8}]\Big (\frac{a}{h}\Big )^{5}. \end{aligned}$$

${\bar{b}}=\frac{b}{a}$ is the normalized slip length. The described correction factor is valid if the particle in the fluid has only a translational movement parallel to a slip wall, such as the case we investigate in this paper^48^.

## Results and discussion

**Figure 1:**
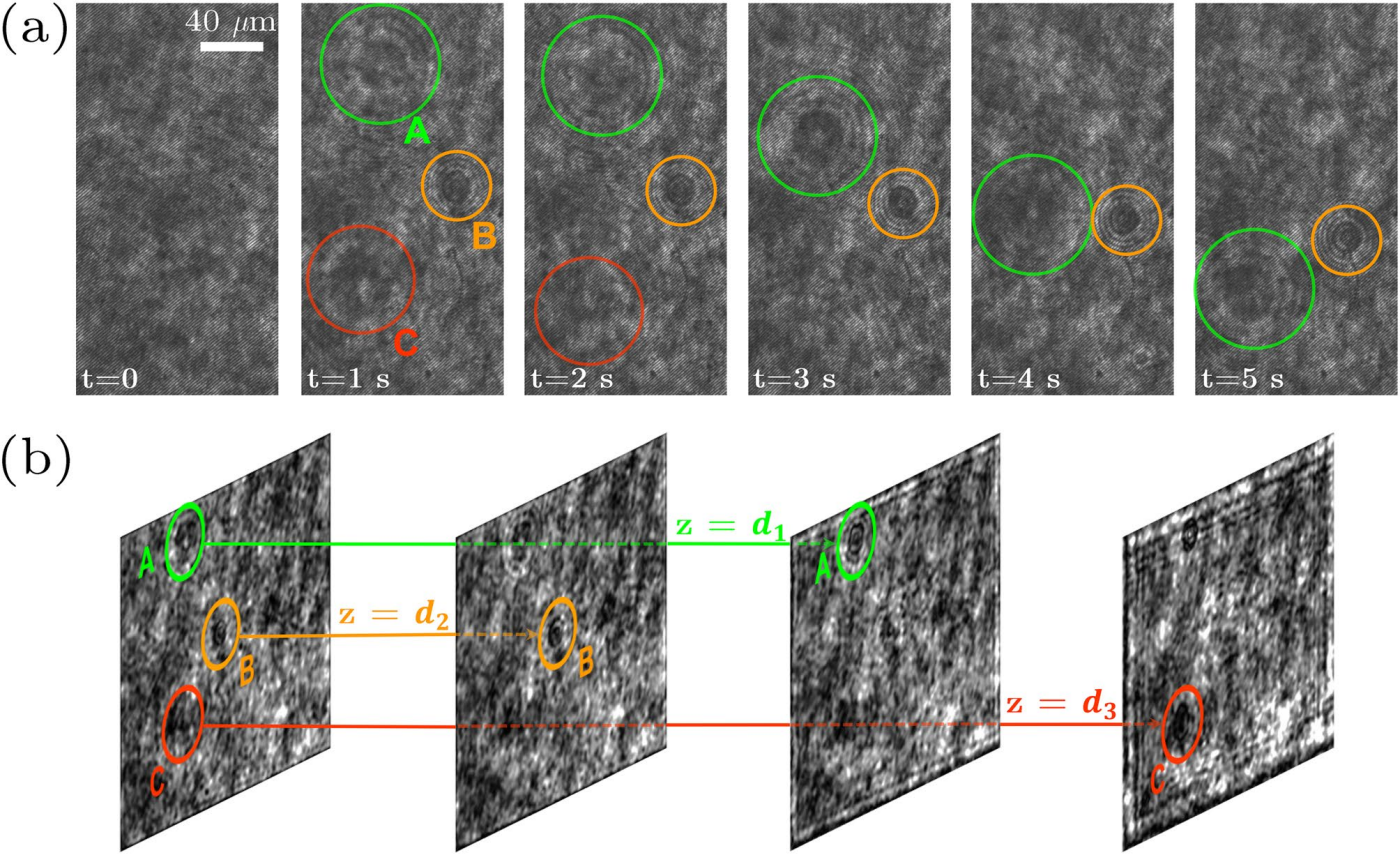
(**a**) The recorded holograms at every 1 s of sedimenting micro-particles in a plastic chamber without a coating for the first 5 s of the experiment. In the first panel, the associated reference hologram is shown, which is acquired in the beginning of the experiment, when no particle is present in the field of view; (**b**) numerical refocusing of three micro-particle in different distances from the flat wall ($d_1=45~\upmu$m, $d_2=60~\upmu$m, $d_3=75~\upmu$m).

In this research, to obtain the depth information of particles falling inside the fluid, we use the numerical refocusing feature of DHM. A micro-particle stuck to the chamber wall is taken as a reference, for which the intensity image is reconstructed when it is in focus. Then, for any particle moving inside the chamber, the numerical refocusing distance that makes the particle’s intensity profile similar to that of the reference one will be the particle-wall distance, *h*. Considering the uniform size and physical characteristics of the polystyrene micro-particles, the cross-sectional profile of a focused image is a sharp one. Therefore, the particles in different depths can be clearly distinguished. In our numerical processing this is taken as the fingerprint of the axial position of a particle. In general, in order to determine the focal plane in numerical focusing of DHM, a focus measure is defined and optimized^41,49^. The lateral information of the particles can be measured by the acquisition of successive digital holograms and knowing the acquisition frame rate and the pixel size in the images. Figure 1 shows the process. Before onsetting the experiment, i.e., dispersing micro-particles inside the quiescent fluid from the upper surface, we acquire a reference hologram. The phase of such hologram, in the reconstruction process, is subtracted from the one of “object” holograms. This, up to a high extent, ensures of removing the effects of possible contaminations and aberrations of optical elements in the optical setup. The holograms in Fig. 1a belong to the reference hologram at $t=0$, and successive sample holograms at *t*=1, 2, 3, 4, 5 s. The numerical refocusing process for three sample micro-particles, A, B, and C, which sediment in different depths is shown in Fig. 1b. $z=d_1, d_2, d_3$ are their corresponding axial distances. Note that only a selection of the field of view is shown in Fig. 1.

**Figure 2:**
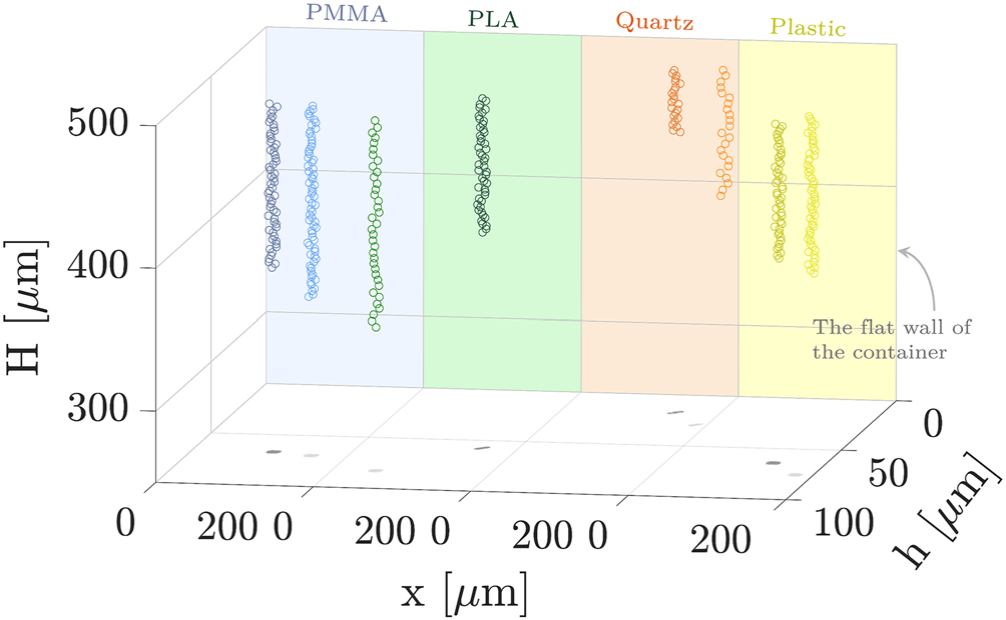
3D trajectories of several polystyrene micro-particles, in different distances from the flat wall of the container within 5 s and in different levels of hydrophobicity wall effects (PMMA, PLA, Quartz, Plastic). The different materials and the sedimenting micro-particles near them are shown in different colors. The different colored circles show the sedimenting micro-particles distances to the flat wall; lighter colors indicate farther ones.

**Figure 3:**
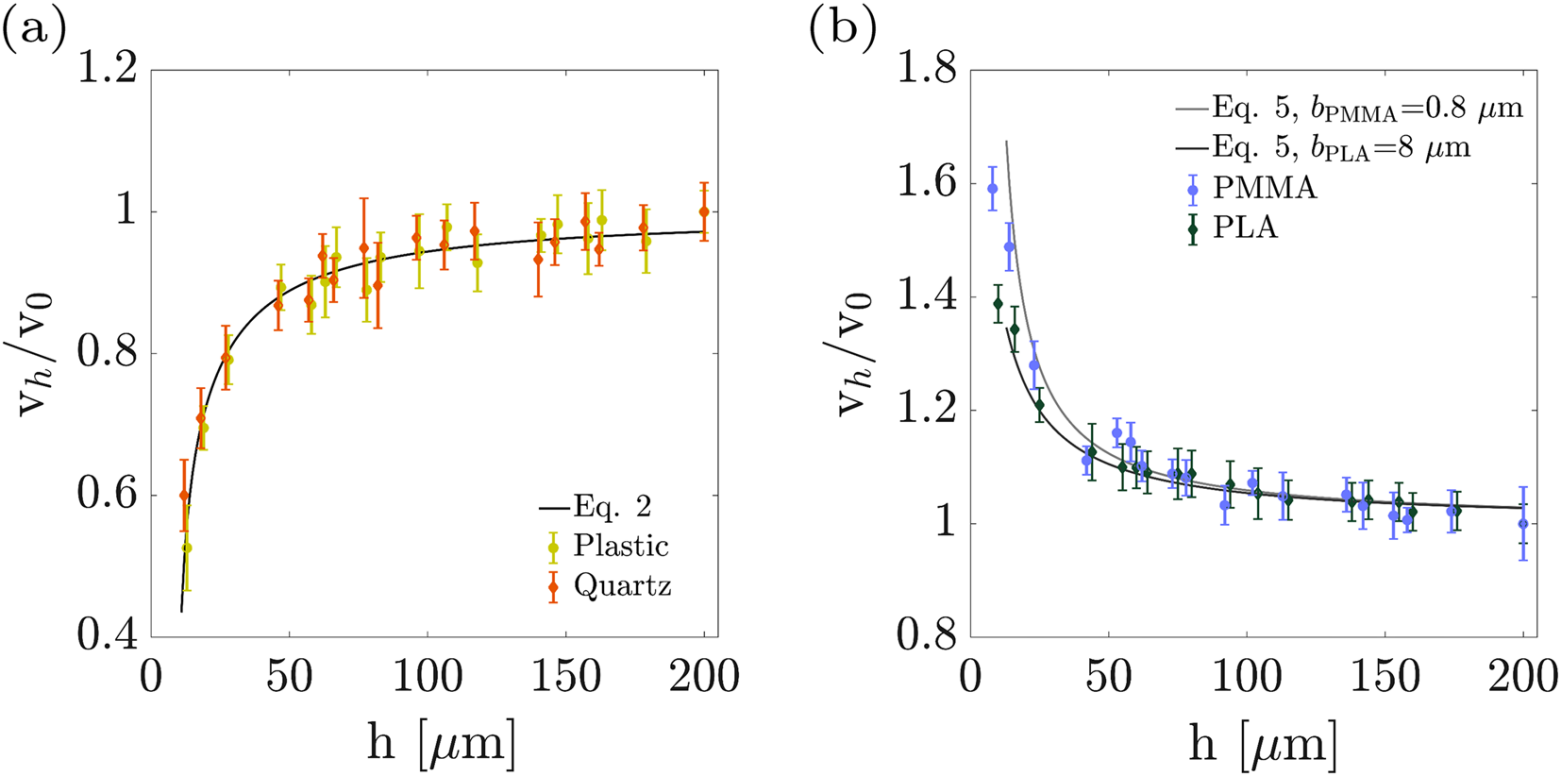
Sedimenting velocity of micro-particles versus their distances from the flat wall in different slip lengths including and the theoretical predictions. (**a**) Plastic and Quartz, which follow Eq. (2) are the no-slip case, and (**b**) PMMA and PLA, which follow Eq. (5) and have the slip lengths of 0.8 $\upmu$m and 8 $\upmu$m, respectively.

We apply the whole procedure for several micro-particles sedimenting in a quartz chamber which has a thin film coating on one of its inner walls. We consider two different coatings, PMMA and PLA, to consider the slip length effect. Moreover, for the comparative study, we repeat the sedimentation experiments for several micro-particles in a quartz chamber as well as in a plastic chamber without a coating. Therefore, by analysis of successive holograms, the 3D trajectories of multiple multi-depth sedimenting micro-particles in four different cases are determined and used to investigate the influence of the proximity to the flat wall of various slip lengths. Figure 2 illustrates the trajectories of typical micro-particles sedimenting in two different distances to the flat wall for the four aforementioned cases. Blue, green, orange, and yellow colors of the wall and the circles correspond to the quartz chamber with PMMA coating, quartz chamber with PLA coating, quartz chamber without a coating, and the plastic chamber without a coating, respectively. Lighter-colored trajectories correspond to farther distances of the micro-particles from the flat wall. This can be better seen through their shadow on the bottom surface. The shadows also confirm that the sedimenting particles have negligible movements in lateral directions and fall down taking an almost vertical trajectory. The micro-particles fall to different heights in the same duration of the experiment due to the different distance from the flat wall. This indicates that, as expected from previous theoretical and experimental studies^10,11,17^, the micro-particles have different sedimentation velocities at different depths due to the proximity effect of the flat wall. More importantly, comparing the quartz results with the coated ones, a considerable difference in the trajectories is evident; polymer coating facilitates the sedimentation process, and the effect is more pronounced for PLA coating that has a longer slip length.

The slip length can be experimentally measured by several methods, such as rheometry and particle image velocimetry^50^. In general, various factors affect the slip length, including surface roughness, surface wetting, cutting speed, nano-bubbles presence, pore radius, liquid polarity, liquid viscosity, temperature, and pressure gradient^51^. These effects, however, cannot be measured individually in the experiments^52^. Instead, there is a relationship between the slip length and the contact angle, and therefore, the liquid-solid molecular interactions, which can be used to estimate the slip length^53,54^. When the wall is hydrophilic, the interactions are strong, and the contact angle is smaller than 90$^{\circ }$, which leads to a smaller slip length^54^. It is predicted that by increasing the contact angle, the slip length increases exponentially^52^. We keep the physical parameters, such as temperature, liquid properties, and pressure constant throughout the experiments, and only the hydrophobicity of an inner wall of the chambers is altered for the investigations. From Wenzel and Cassie-Baxter equations, the contact angle is also strongly dependent on the wall roughness^55,56^. When the contact angle is below 90$^{\circ }$, the roughness makes the effective contact angle smaller, and thus the slip length decreases, and above 90$^{\circ }$ by increasing the roughness due to the presence of trapped gas on a rough wall^57,58^.

The experimental results of the flat wall effect on micro-particles sedimentation are demonstrated in Fig. 3, and the variations of relative sedimenting velocities as a function of particle-wall distance are measured. Each data point in Fig. 3 is the average of measurements on an ensemble of at least 10 micro-particles. Figure 3a shows the variations of velocities in the proximity of a flat wall in a no-slip regime in plastic (yellow-green circles) and quartz (dark-orange diamond) cases. The results for both cases are in good agreement with the theoretical predictions (Eq. 2), shown by the black solid line. Figure 3b demonstrates the sedimenting velocities of micro-particles at different distances from the PMMA (blue circles) and PLA (dark-green diamonds) coated flat walls, which have different slip lengths. Indeed, according to Eq. (5), sedimenting experiments near the wall and at different distances provide, additionally, a slip length measurement approach. Our experimental results are in a very good agreement with the theoretical prediction (gray and black lines for PMMA and PLA cases, respectively). By fitting the function of Eq. (5) to the experimental data, the slip length of PMMA and PLA are measured as 0.8 $\upmu$m and 8 $\upmu$m, respectively.

**Figure 4:**
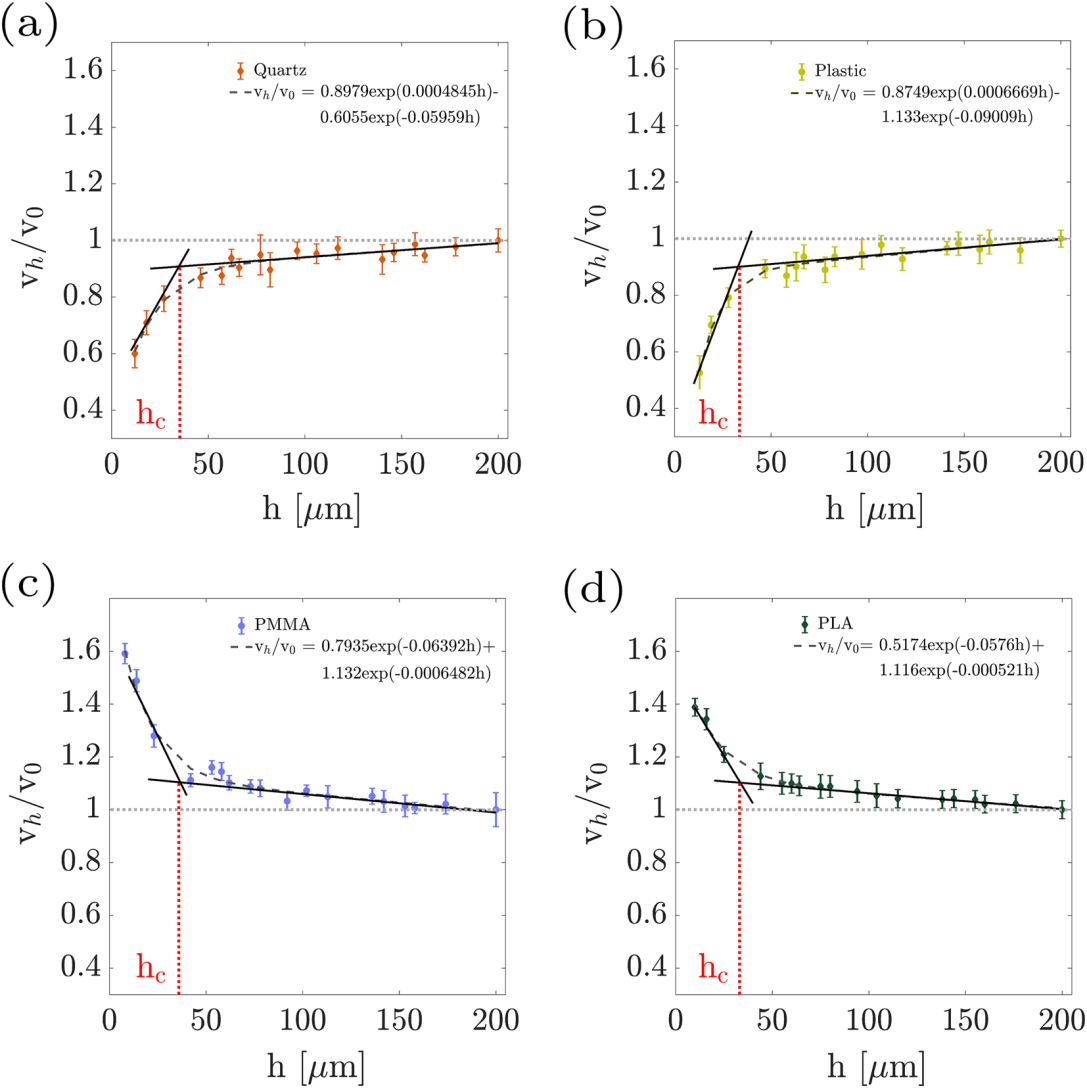
Obtaining the characteristic distance, $h_c$, which is a measure for the sensitivity of the sedimentation process to the wall presence. This value is obtained from the intersection of fitted lines to the $\frac{v_{h}}{v_{0}}$ values at small and large distances to the flat wall. (**a**) PMMA; (**b**) PLA; (**c**) Quarts; (**d**) Plastic.

**Figure 5:**
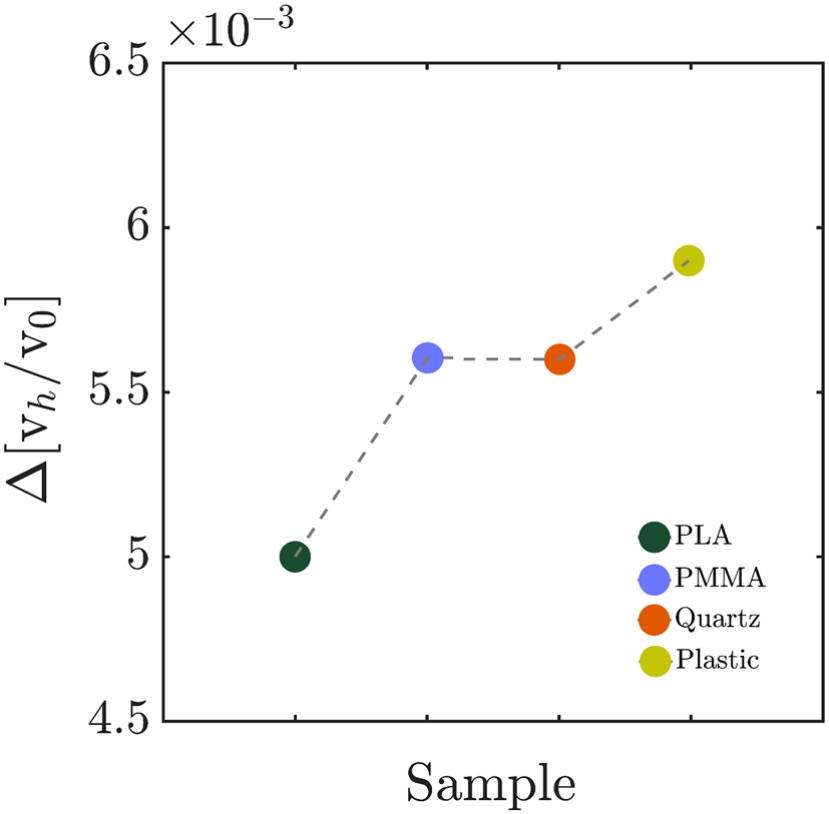
Slope of wall factor variations, $\Delta$[$\frac{v_{h}}{v_{0}}$], of sedimenting micro-particles for the flat walls of different slip lengths.

In both no-slip and slip cases, two distinguishable regimes may be detected. Initially, the sedimentation velocity has a dramatic variation as a function of particle-wall distance, and at farther distances, it reaches a plateau. We pursue the sensitivity to the wall presence in each case by considering a characteristic distance, $\mathrm {h_c}$. This value is obtained from the intersection of fitted lines to the $v_h/v_0$ values at small and large distances to the flat wall. The process is shown in Fig. 4 for all the studied sample chambers. The fitted functions, following the Eqs. 2 and 5 for no-slip and slip cases, respectively, are also presented in the insets of Fig. 4a–d. In Fig. 5, we compare the rates of reaching to plateau for the studied cases. This parameter, actually, is the derivative of the flat-wall proximity correction factor, $\Delta [\frac{v_h}{v_0}]$ at the characteristic distance. The experimental results show that the effect of the presence of PLA coated wall on the sedimentation of micro-particles vanishes faster than the other cases. In general, the influence of flat walls of different slip lengths can be interpreted by consideration of continuous particle translation in both transverse and perpendicular directions, which is very recently predicted through a rigorous theoretical model due to the presence of surface roughness^59^.

These observations might be useful in manipulating and controlling of any sedimenting phenomenon in industrial and life science and technology. Essentially, any phenomenon in the presence of boundaries, such as the active motion of biological micro-swimmers near boundaries and motion of colloidal particles in microfluidic channels, is included in this class. Furthermore, the dependence of the quantifiable parameter to the boundary properties, e.g., sedimenting velocity and distance from the boundary, may provide a noninvasive approach for measurement of surface properties in microrheology.

In conclusion, we measured the proximity effect of the wall on the sedimentation of colloidal microspheres near the surfaces with different slip lengths by the use of digital holographic microscopy (DHM). DHM provides quantitative phase-contrast and 3D imaging in arbitrary time scales, which makes it a suitable method to investigate various phenomena, including the dynamic behavior of colloids in 3D^22–24,60–62^. We have observed that for lower slip length values, the proximity effect is more pronounced, while the falling velocity variation of the particles increases by the slip length of the wall. Sedimenting phenomenon is ubiquitous in nature, and most of them also include the presence of boundaries, such as the motion of biological micro-swimmers near boundaries and motion of colloidal particles in microfluidic channels. The results of this research might have usefulness in such studies. It may also provide a noninvasive approach for the measurement of surface properties in microrheology. The capability of the presented methodology for 3D imaging and tracking has the potential to be used for different applications in the study of colloidal dynamics where 3D information in real-time and arbitrary time-scales is required.

## Methods

### Sample preparation

#### Sedimenting particles

We used polystyrene microspheres with a diameter of 20±1 $\upmu$m as a sample. The micro-particles are diluted in distilled water, and this dilution is such that the hydrodynamic interactions of the particles with each other can be avoided, as well as micro-particles that are close to the wall at different distances and in the field of view of the imaging system will be limited. In each stage of the experiment, a 2 $\upmu$l droplet of the prepared sample is added at a close distance to the wall (approximately 1 mm) to the vertically stood chamber filled up by distilled water.

#### Sample chambers

The sample chambers are 3.5 ml cuvette cells with a 10 mm $\times$ 10 mm cross-section and 30 mm length. We use two cells made of quartz (Spectrocell, Q124) and plastic (Biofil, CUV010045). Polylactic acid (PLA, MW = 60000) and poly(methyl methacrylate) (PMMA, MW = 300000) are purchased from Sigma-Aldrich. Sodium sulfate and Dichloromethane (DCM) are obtained from Merck. DCM is distilled over sodium sulfate before any use.

**Figure 6:**
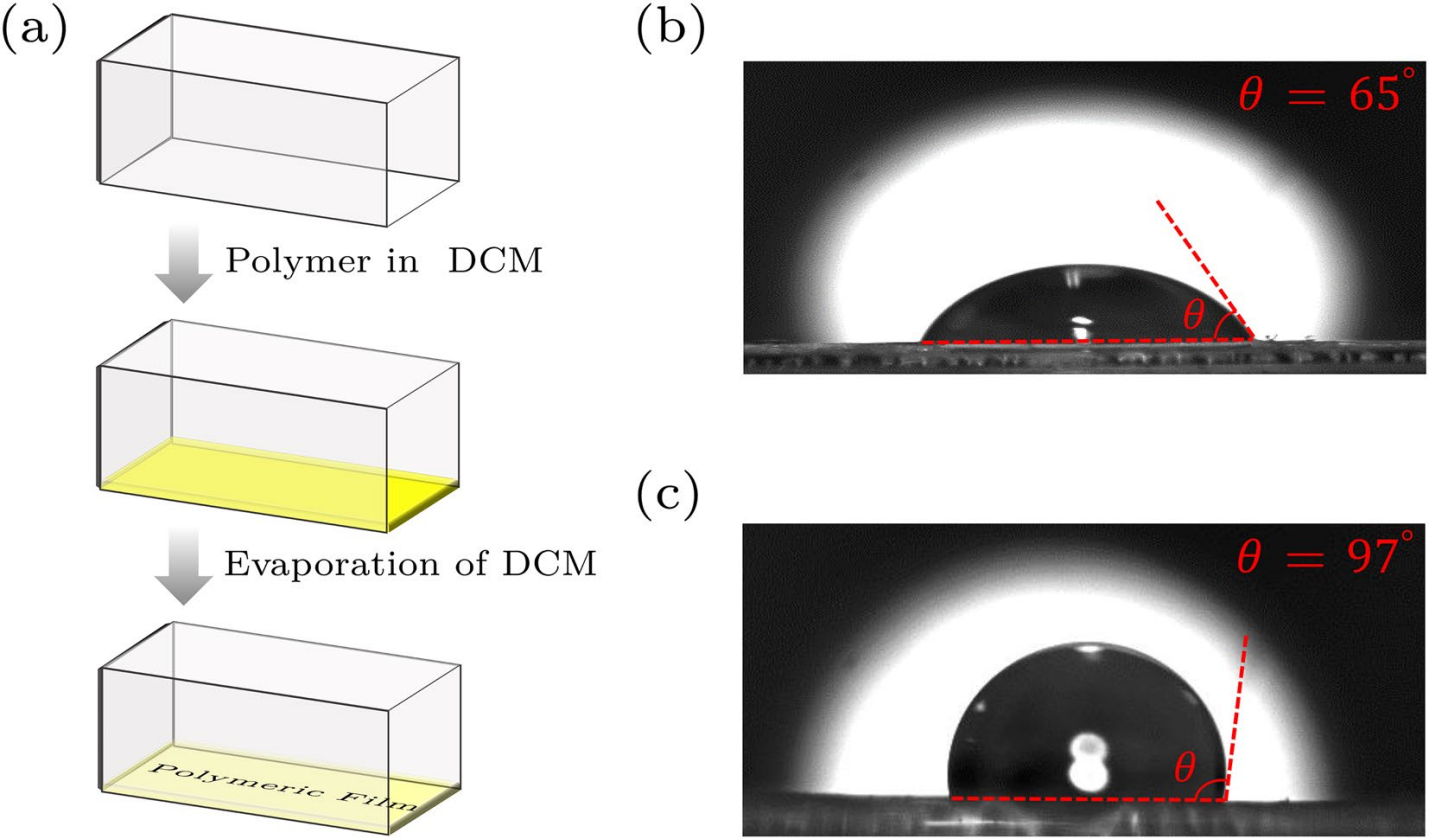
(**a**) Polymer coating procedure; (**b**,**c**) Contact angle measurement of PMMA and PLA polymers, respectively.

#### Film preparation

Different fabrication techniques have been developed for the preparation of polymeric thin films on a given substrate. Amongst, solvent-casting approaches have been extensively used, because of the easy control of film thickness in such approaches and their cost and simplicity. In this research, PLA and PMMA files inside the sample, chambers are prepared using this method. We first add 100 mg of PMMA or PLA to 3 ml of distilled DCM and stir it for several minutes to obtain a homogeneous solution. Then, while the chamber is being flipped horizontally, using a syringe 1 ml of the prepared solution is poured into the chamber. Upon evaporation of DCM, the film remains coated over one of the inside walls of the chamber. The procedure is shown schematically in Fig. 6a.

#### Contact angle measurement

Static water contact angle (CA) measurements were performed at 24 ± 2 $^{\circ }$C by placing a droplet of deionized distilled water on the surfaces. The acquisition of images from the open side of the chamber is done using a digital camera (Thorlabs, 8-bit dynamic range, 5.2 $\upmu$m pixel pitch) and a mounted 12 mm lens. The contact angle is measured by post-processing the acquired images in a computer. The experiments are repeated 10 times for each sample, and the average CA is reported. For the PMMA and PLA surfaces, the CAs of the static water are obtained 65$^{\circ }$ and 97$^{\circ }$, respectively (Fig. 6b,c).

**Figure 7:**
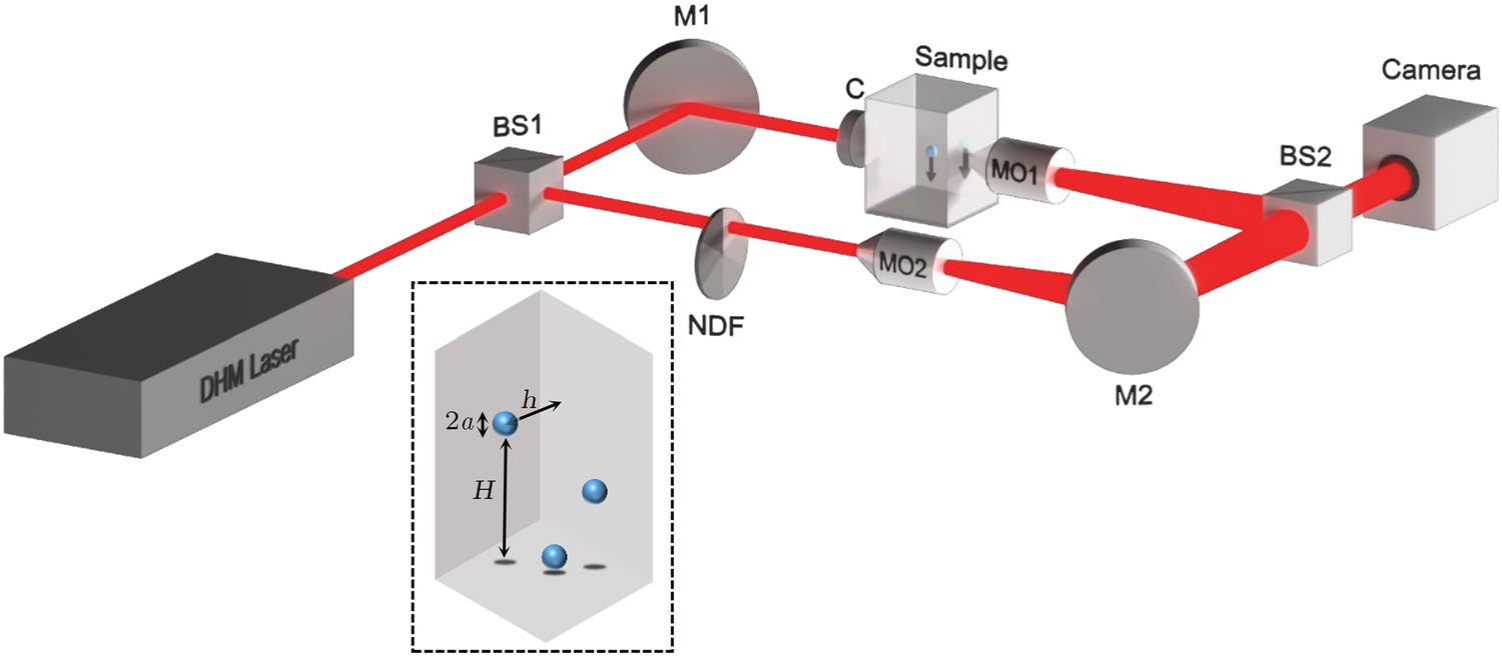
The schematic experimental setup; BS: beam splitter, NDF: neutral density filter, MO: microscope objective, M: mirror, C: condenser. Inset: enlarged view of the sample chamber; *a*: micro-particle radius, *h*: micro-particle distance from the chamber flat wall, *H*: vertical position of micro-particle.

### Experimental procedure

The DHM system transmission mode based on the Mach-Zehnder interferometer is used in this research. The schematic experimental setup is shown in Fig. 7. The output from a laser source (He–Ne Laser, 632.8 nm, 5 mW) is split into two identical beams using a 50 : 50 beam splitter (BS1). One of the beams, called the object beam, is directed by a mirror (M1) and a condenser (C) into the sample to illuminate it. The scattered light from the sample is collected by a 40x microscope objective (MO1, Olympus, NA = 0.65, WD = 0.17 mm) and is sent onto the digital camera (Thorlabs, 8-bit dynamic range, 5.2 $\upmu$m pixel pitch) through a second beam splitter (BS2). The other beam, which is the reference beam, reaches the recording camera passing through M2, MO2, and BS2, and interferes with the object beam to create a digital hologram. In order to attain off-axis geometry for DHM and to avoid the overlap of the reference and the twin images, a slight angle applied between the reference and the object beams. MO2 in the reference arm of the setup is identical to MO1 and is used to match the curvature of the reference wavefront with the one of the object beam. A neutral density filter (NDF) is also used in the reference arm to match the intensities of the object and reference beams. The insertion of MO2 and NDF ensures the formation of appropriate interferometric fringes in terms of linearity, uniformity, contrast, and density. The digital holograms are recorded at 10 fps speed during the process of sedimentation.

In the inset of Fig. 7 a magnified scheme of a sedimenting particle inside the chamber is shown, and the parameters *h* and *H*, which are the distances of the particle from the inner flat wall and the bottom surface of the chamber, respectively, are shown.

### Numerical processing

The procedure of DHM consists of two steps: recording and reconstruction. The first step, i.e., recoding of a hologram, is similar to the conventional holography, except that the interference pattern of the object and reference beams are formed and acquired on a digital sensor such as a CMOS or CCD camera. However, the main advantage of DHM over conventional holography is in the reconstruction stage, in which the recorded hologram is subjected to numerical processing in a computer toward extracting the full field distribution of the holographic image and further 3D and morphometric information therefrom. The reconstruction process is done by simulating the diffraction from the digital hologram when illuminated by the reference wave, followed by numerical propagation into the desired image plane. It leads to the complex amplitude, from which the amplitude and the phase information can be extracted. The number of approaches have been presented for numerical reconstruction of digital holograms, such as Fresnel transform, Huygens convolution, and angular spectrum propagation approach (ASP)^63^. In this research, we use the ASP approach. In the reconstruction process, once the recorded digital hologram is illuminated by the reference beam, upon diffraction, it forms a virtual image, a real image, and zero-order terms. In ASP, due to dealing with Fourier space, it is straightforward to filter out the unwanted terms. In the following, we present the details of the reconstruction approach and its possibility to provide 3D tracking information of multiple sedimenting micro-particles from a single recorded hologram.

The complex wavefield, *E*(*x*, *y*, 0), is obtained through numerical illuminating the digital hologram by the reference wave at the hologram’s plane, (*x*, *y*, 0), and the angular spectrum is defined by the Fourier transform:

8 $$\begin{aligned} {\tilde{E}}(x,y,0)=\int \!\!\!\int _{-\infty }^{+\infty } E(x,y,0) e^{-i2\pi (ux+vy)} \, dx \, dy, \end{aligned}$$

where *u* and *v* are the spatial frequencies in the *x* and *y* directions, respectively, and “$\sim$” denotes Fourier transformation. In the Fourier plane, according to the off-axis arrangement, the spatial frequencies associated with real and virtual images and the zero-terms are separated. Indeed, the proper adjustment of the interfering fringes during the recording stage is significant so that, on the one hand, the overlap of spatial frequencies of different terms is avoided, and on the other hand, their distances are not larger than the size of Fourier window. Moreover, further improving processes such as the smoothing process can be performed in this stage. By filtering the resulting spectrum, the information about the object is selected and is brought into the center of the Fourier coordinates. The complex amplitude at the image plane, which is located at distance *d* from the hologram plane, is obtained by free-space propagation of ${\tilde{E}}^{F}(u,v,0)$ in the Fourier space and performing an inverse Fourier transform:

9 $$\begin{aligned} E^{F}(x,y,d)=\int \!\!\!\int _{-\infty }^{+\infty } {\tilde{E}}^{F}(u,v,0) e^{ikz \sqrt{1-\lambda ^{2}u^{2}-\lambda ^{2}v^{2}}} \times e^{i2\pi (ux+vy)} \, du \, dv, \end{aligned}$$

where $\lambda$ and *k* are the laser wavelength and the wavenumber, respectively, and $\{...\}^{F}$ represents the filtering process in the Fourier domain. The whole process can be written as the following expression:

10 $$\begin{aligned} E^{F}(x,y,d)=FT^{-1} \bigg \{ \Big [FT \big \{ E(x,y,0) \big \}\Big ]^{F} e^{ikd \sqrt{1-\lambda ^{2}u^{2}-\lambda ^{2}v^{2}}} \bigg \}, \end{aligned}$$

where *FT* and $FT^{-1}$ represent 2D Fourier and inverse Fourier transforms, respectively. From the complex amplitude of the object wavefront, the intensity of the image (*I*), as well as its phase ($\phi$), can be computed:

11 $$\begin{aligned}I(x,y,d)=\big | {\tilde{E}}^{F}(x,y,d) \big |^{2}, \end{aligned}$$

12 $$\begin{aligned} \phi (x,y,d)= \arctan
\frac{{{\mathrm{{Im}}}[{\tilde{E}}^{F}(x,y,d)]}}
{{{\mathrm{{Re}}}[{\tilde{E}}^{F}(x,y,d)]}}.
\end{aligned}$$

Equation (11) provides image intensity that is similar to a conventional microscope image, and 3D information of the object is obtained from Eq. (12). The phase obtained from Eq. (12) takes values between $-\frac{\pi }{2}$ and $\frac{\pi }{2}$, which creates a discontinuity throughout the phase map. The discontinued phases are converted to continuous phase maps by an unwrapping method, such as Goldstein’s branch-cut unwrapping algorithm^64^. The phase is proportional to the optical path length, $\phi =\frac{2\pi }{\lambda }nL$, where *n* is the refractive index and *L* is the physical length that the light beam passes through.

An important advantage of DHM over conventional microscopy is numerical refocusing. It means that in the reconstruction process, by varying the parameter *d*, it is possible to change the image formation plane. This is the key feature that is incorporated in our research to track the sedimenting particles. A digital hologram of a collection of sedimenting particles in the field of view is acquired. Particles of different distances to the chamber wall will be in different focuses. By playing with *d* and formation of the focused images of any of the particles, indeed, their distances to the flat wall will be achieved. Acquisition and reconstruction of successive digital holograms will, therefore, lead to obtaining the 3D trajectories of multiple sedimenting particles. Their velocities are also obtained by considering the recording frame rate and the sedimentation duration time. This feature of DHM, along with the possibility to acquire phase images, makes it a unique methodology to apply in several phenomena in microfluidics, as it provides complete space and time information of the dynamic process^65^. To remove the effects of the background contaminations and the aberrations of optical elements from the final data, before the onset of the experiment, a reference hologram is recorded. To this end, the chamber filled up with distilled water is used. The reconstructed phase of the reference hologram is then subtracted from the other recorded holograms.
